# Development and Local Contextualization of Mobile Health Messages for Enhancing Disease Management Among Community-Dwelling Stroke Patients in Rural China: Multimethod Study

**DOI:** 10.2196/15758

**Published:** 2019-12-17

**Authors:** Enying Gong, Wanbing Gu, Erdan Luo, Liwei Tan, Julian Donovan, Cheng Sun, Ying Yang, Longkai Zang, Peng Bao, Lijing L Yan

**Affiliations:** 1 Global Health Research Center, Duke Kunshan University Kunshan China; 2 School of Population and Global Health, The University of Melbourne Melbourne Australia; 3 Institute of Chinese Medical Sciences, University of Macau Taipa, Macau China; 4 School of Public Health, Fudan University Shanghai China; 5 Northumbria Healthcare National Health Service Foundation Trust Wallsend United Kingdom; 6 Second School of Clinical Medicine, Wuhan University Wuhan China; 7 Ningxia Medical University Yinchuan China; 8 Duke Global Health Institute Durham, NC United States

**Keywords:** phone messages, stroke, secondary prevention, rural population, China

## Abstract

**Background:**

Rural China has experienced an increasing health burden because of stroke. Stroke patients in rural communities have relatively poor awareness of and adherence to evidence-based secondary prevention and self-management of stroke. Mobile technology represents an innovative way to influence patient behaviors and improve their self-management.

**Objective:**

This study is part of the System-Integrated Technology-Enabled Model of Care (the SINEMA trial) to improve the health of stroke patients in resource-poor settings in China. This study aimed to develop and pilot-test a mobile phone message–based package, as a component of the SINEMA intervention.

**Methods:**

The SINEMA trial was conducted in Nanhe County, Hebei Province, China. A total of 4 villages were selected for pretrial contextual research and pilot study. The 5 stages for developing the mobile phone messages were as follows: (1) conducting literature review on existing message banks and analyzing the characteristics of these banks; (2) interviewing stroke patients and caregivers to identify their needs; (3) drafting message contents and designing dispatching algorithms for a 3-month pilot testing; (4) collecting feedback from pilot participants through questionnaire survey and in-depth interviews on facilitators and barriers related to their acceptance and understanding of messages; and (5) finalizing the message-based intervention based on participants’ feedback for the SINEMA trial.

**Results:**

On the basis of 5 existing message banks screened out of 120 papers and patients’ needs identified from 32 in-depth interviews among stroke patients and caregivers, we developed a message bank containing 224 messages for a pilot study among 54 community-dwelling stroke patients from 4 villages. Of 54 participants, 51 (response rate: 94.4%) completed the feedback survey after receiving daily messages for 3 months. Participants’ mean age was 68 years (SD 9.2), and about half had never been to school. We observed a higher proportion of participants who were in favor of voice messages (23/42, 54%) than text messages (14/40, 35%). Among participants who received voice messages (n=43) and text messages (n=40), 41 and 30, respectively, self-reported a full or partial understanding of the contents, and 39 (39/43, 91%) and 32 (32/40, 80%), respectively, rated the messages as helpful. Analyses of the 32 interviews further revealed that voice messages containing simple and single-theme content, in plain language, with a repeated structure, a slow playback speed, and recorded in local dialect, were preferred by rural stroke patients. In addition, the dispatching algorithm and tools may also influence the acceptance of message-based interventions.

**Conclusions:**

By applying multiple methodologies and conducting a pilot study, we designed and fine-tuned a voice message–based intervention package for promoting secondary prevention among community-dwelling stroke patients in rural China. Design of the content and dispatching algorithm should engage both experts and end users and adequately consider the needs and preferences of recipients.

## Introduction

### Stroke Secondary Prevention in Rural China

Stroke is the leading cause of death and disability in China. According to recent estimates, stroke-related deaths reached around 1.1 million in China, accounting for 23% of total deaths in rural areas and 21% in urban areas [1]. In addition, it is estimated that there are 12.4 million stroke patients aged older than 40 years living in China, which causes an enormous burden to the health care system and society [2]. Compared with urban areas, rural China has experienced a more rapidly increasing burden, with the age-standardized prevalence of stroke increasing 2.5-fold over the past three decades [3].

Stroke patients in rural areas have relatively low awareness of and poor adherence to the secondary prevention and self-management of stroke. Effective secondary prevention of stroke, including lifestyle modification and a combination of medical therapy (eg, antiplatelet, antilipid, and antihypertensive therapy), has been well studied as the *best buy* for stroke patients [4]. However, adherence to these effective preventive strategies is poor among stroke patients, with more than half of patients reported discontinuing their secondary prevention medications within 3 months of hospital discharge [5,6]. In addition, the fragmented primary health care system and limited capacities of health care providers in rural China further restrain the availability and quality of the health care services that stroke patients can gain access to [7]. Therefore, there is an urgent need to develop a low-cost and effective strategy to increase the awareness of and adherence to secondary prevention measures of stroke in rural China.

### Message-Based Intervention

With the development of mobile communication technology and widespread adoption of mobile phones, mobile health (mHealth) has the potential to empower patients to improve their self-management of chronic conditions and related risk factors [8,9]. A previous literature review illustrated that compared with high-income countries where more advanced mobile technologies are used, low- and middle-income countries have relied more on phone messages for intervention delivery because of its accessibility, lower cost, and better acceptance [10]. Review studies and meta-analysis have illustrated the modest effect of message-based interventions in promoting medication adherence and lifestyle modifications and improving health outcomes among patients with chronic conditions, although further studies with longer follow-up period and larger scale were suggested [8,9]. The evidence on message-based studies targeted at stroke patients was very limited, with a few studies focused on recently discharged stroke patients [11], but almost none on community-dwelling patients. In addition, there were only a few studies that tried to understand the feasibility and impact of message-based interventions among patients with cardiovascular diseases in China, and most of these studies focused on urban patients [12]. The acceptability and feasibility of applying message-based interventions among rural patients still need to be further investigated.

In addition, some of the previous reviews attempted to examine why some of the message-based interventions work or do not work. Factors related to message contents and dispatching algorithms, such as message frequency, content personalization, and dispatching approach in 2 ways, were examined, but the results were mixed [13,14]. Studies also pointed out that lacking theoretical constructs may lead to the difficulty in understanding the mechanisms behind the message-based interventions in chronic disease management. Watkins et al conducted a realist review by mapping the intervention components with psychological theories and frameworks [15]. The authors found that information provided via messages to increase patients’ knowledge could motivate patients to believe the relevance of the issue to their health and the potential risk, which may reduce the threat as suggested by the health belief model [16]. Some other psychological behavior change theories, such as the transtheoretical model or a taxonomy model of behavior change techniques, including goal settings and self-regulation, were also applied in the design of messages [15,17].

### Objectives

This study is part of the System-Integrated Technology-Enabled Model of Care (the SINEMA trial) to improve the health of stroke patients in resource-poor settings in China. The protocol of the SINEMA trial has already been published [18]. This study aimed to describe the development and contextualization process of this message-based intervention package from field-based research and pilot studies. The results of the effectiveness of the message-based intervention in the SINEMA main trial is beyond the scope of this paper.

## Methods

### Study Setting

The SINEMA trial and this pilot study were conducted in Nanhe County, Hebei Province, China, with an intention to be adapted to other resource-limited settings. Nanhe County is an economically poor provincial county with an annual disposable income per capita of 11,030 RMB (less than half of the national average) [19]. Although no specific estimation on stroke burden is available in this county, previous studies have shown a high burden of stroke in this province and rural settings compared with other parts of China [20].

To ensure the study design and blind generalization of the intervention model, we aimed to identify 4 villages out of 218 villages within the geographical area to implement the field-based contextual research and pilot study, and these 4 villages were not eligible to be included in the trial. We received assistance from the local Center for Disease Control and Prevention to identify 4 villages where village doctors were willing to participate. These 4 villages comprised 2 villages from a township that had an insufficient number of villages to be eligible for the main trial and 2 villages from eligible township but with insufficient residents to meet the inclusion criteria of the main trial. Therefore, we were able to identify typical villages but also leave the potential eligible large villages for the main trial.

### Message Design Process

**Figure 1:**
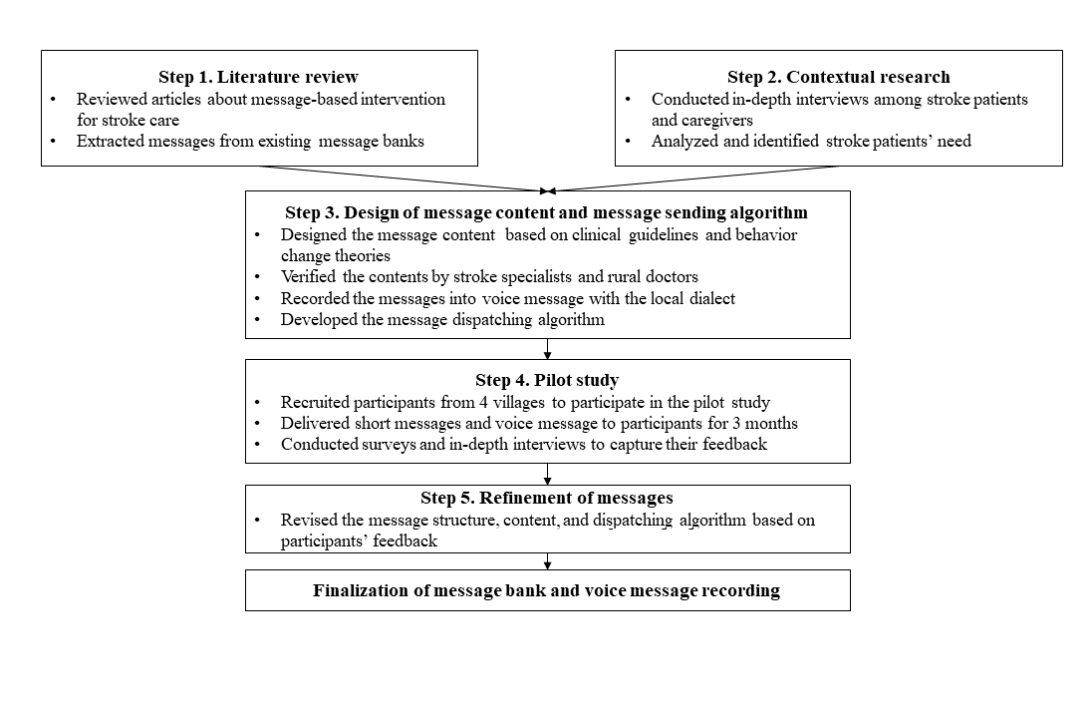
Overview of key stages of message development.

We used multiple methodologies, including literature review, expert consultation, qualitative in-depth interviews, and field-based pilot study followed by surveys and interviews, in developing the messages to ensure that the message contents were built based on research evidence and in line with the clinical guidelines and were suitable for the local context. The development process for the mobile phone message banks consisted of the following 5 stages: (1) conducting a literature review on existing message banks targeting people with stroke, (2) interviewing stakeholders to identify the needs of stroke patients, (3) creating and designing the message contents and message sending algorithm, (4) conducting pilot testing of the messages among patients in 4 villages, and (5) refining and finalizing the message bank and sending algorithm based on the lessons learned from the pilot study. Figure 1 outlines the design process.

#### Stage 1. Literature Review on Existing Message Banks

To assist the design of the message bank, we conducted a literature review based on the PubMed database to search for existing text message banks published between October 1, 2011, and October 1, 2016. Our own design of the messages began in November 2016. Key search terms included stroke and text messages (for search terms and review criteria, see Multimedia Appendix 1). A total of 2 reviewers screened the searched papers, and a snowball review by reviewing titles and abstracts of references of searched papers was also conducted to increase the scope of the review. Existing message banks were extracted and translated into Chinese as a reference for the design.

#### Stage 2. Contextual Research

To identify the needs of stroke patients, we visited the field 3 times over the period of 5 months from May to September 2016 and conducted in-depth interviews among stakeholders, including stroke patients and family caregivers. Semistructured interview guides were developed, and questions in the interview included patients’ health status and needs for managing their conditions. Stroke patients were identified with the assistance of village doctors with the criteria that the participants were aged 18 years or older, had a history of stroke diagnosed at the county- or higher-level hospitals, were in clinically stable condition, had basic communication abilities, and were willing to participate in the study. Stroke patients’ family members who were at home while the research team visited and were willing to participate were also interviewed separately. The number of participants interviewed was determined based on the saturation theory, whereby participants were recruited until no further new information was acquired through the interview process. We expect to interview about 4 patients from each village.

#### Stage 3. Design of Message Content and Message Sending Algorithm

We designed the contents of messages on the basis of the existing message banks from the literature review, the identified needs of stroke patients from contextual research, and the behavior change theories such as the health belief model and the transtheoretical model [16,21]. First, the structure and focus of the message bank were identified so that messages could tap into key dimensions based on patients’ needs. We then grouped the existing messages into 6 categories based on their contents, including management of metabolic risk factors, medication adherence, tobacco and alcohol control, dietary change, exercise and rehabilitation, and psychological support. The content of the messages was modified to be suitable for stroke patients in rural China. The messages were then verified by stroke specialists working in tertiary hospitals and recorded in the local dialect.

We also designed the message dispatching algorithm by setting the sending time and frequency based on the daily habits of stroke patients. This algorithm was linked with a digital health management system that was designed to support the delivery of the digital health components of SINEMA intervention.

#### Stage 4. Pilot Study

A pilot study was conducted in 4 villages to test the SINEMA intervention model, including the acceptance of the message-based intervention among stroke patients. Participants who were aged older than 18 years, had a history of stroke but in a clinically stable condition, and able to communicate via mobile phone were eligible to participate in the study. In each village, village doctors screened stroke patients in their villages and provided the list to the study team. Participants were invited by village doctors and recruited by the research team. Before the commencement of the pilot study, a structured questionnaire, including questions on participants’ demographic characteristics and disease history, designed based on previous studies [22,23], was administered by the research team through face-to-face interviews.

During the 3-month pilot study, participants and their caregivers received text messages at 3 pm every 2 days, and the participants also received voice messages with the same content at 7 pm on alternating days when they did not receive the text messages. At the end of the pilot study, participants completed a short questionnaire administered by their village doctors. Questions in the survey included whether they had read or listened to the messages, their understanding of the contents of the messages, and their perspectives on the helpfulness of these messages. In-depth interviews were also conducted by the research team among selected participants to seek detailed feedback.

#### Stage 5. Refinement of the Messages

After the pilot study, the research team summarized the feedback from participants and refined the message contents and message dispatching algorithm. To optimize the acceptance and understanding of the messages, the research team revised the language in each message based on participants’ feedback and preference. Village doctors and physicians from county hospitals were invited to verify the messages to ensure that local contexts were taken into full consideration, and the terms used in the messages were understandable by the target population.

### Data Analysis

To apply the interview results to message development, we analyzed the interview transcripts using thematic analysis approach, which is a method for identifying, analyzing, and reporting patterns within data and is able to both reflect the reality and explain the undersurface meaning [24]. Following the thematic analysis approach, we conducted the following steps: the research team first read the transcripts of the interviews to be familiar with the data, then generated the key concepts for coding based on the interview guide. Data were coded line by line and grouped into categories using NVivo 11 software (QSR International). Each category of coding was then further reviewed and examined. The groups of key themes were refined before finalizing the major themes. Discussions within the research team were organized after each key step to ensure the trustworthiness and define the final themes. Quotations used in this paper were translated from Chinese to English and then back translated into Chinese to increase the accuracy of the interpretation.

A descriptive analysis was conducted for survey data. Continuous variables were reported as mean and SD if normally distributed or as median with 25th and 75th percentiles if not. Counts and proportions were reported for categorical variables. Survey data were analyzed using STATA software (version 15; StataCorp LLC).

### Ethical Considerations

All research activities, including both contextual research and pilot study, were approved by the institutional review board of Duke Kunshan University. All participants in the study provided informed consent before taking part in the study.

## Results

### Summary

In this section, we first describe the main findings from stages 1, 2, and 4 explained above: the literature review, contextual research, and the pilot study. We then describe how we developed and refined the message bank based on these findings (stages 3 and 5). Finally, we briefly introduce the actual -message-based intervention adopted in the trial.

### Message Bank Review and Extraction

After the systematic search and snowball review of cited papers, we found 5 existing SMS banks. Using these 5 completed SMS banks, we translated messages into Chinese, resulting in a total of 224 messages as the basis for further design. Multimedia Appendix 1 shows the Preferred Reporting Items for Systematic Reviews and Meta-Analyses diagram of the literature review and the contents and features of these 5 identified message banks. 

### Results From Contextual Research on Stroke Patients’ Needs

During the 3 contextual research visits, we interviewed 22 stroke patients and 10 caregivers. Through analyses of these interviews, we identified the major needs of patients and the required contents of messages.

#### Content 1: General Knowledge About Stroke Secondary Prevention

Although almost all patients involved in the interview expressed a strong desire to recover from stroke, most of them had limited awareness and knowledge of secondary prevention of stroke, which was insufficient and not comprehensive enough to help them overcome all challenges that they faced in managing their conditions. There were very few sources available to patients regarding evidence-based information related to stroke secondary prevention:

No physicians told us what we should pay attention to when I was in the hospital. They [physicians] did not care about it. Only the village doctor told us some [information].

Patient #8

Village doctors, considered as the key source of information and the first contact of care, also had the minimal capacity to provide intensive health education to patients because of their existing high workload of serving more than 1000 residents in the village. Therefore, general knowledge related to stroke secondary prevention was required in the message bank.

#### Content 2: Promoting Adherence to Guideline-Recommended Activities

In the interview, many patients admitted that they were not able to adhere to all secondary prevention medications. The main reasons for nonadherence to medication use included their forgetfulness because of declining memory, impaired mental function, or busy working lives. We found that most of the patients we interviewed lived alone or with their spouse alone, without any other caregivers to remind them to take their medications:

I often forgot to take medicine. He [the patient’s husband] usually put all the medicines on my table and reminded me to take them...If he also forgot, we would forget about it totally.

Patient #11

Considering the lack of reminders and the suboptimal adherence among most stroke patients, we decided that the message should act as a reminder for patients to promote their adherence to treatment.

#### Content 3: Providing Specific Guidance on Recommendations for Physical Activity

Through the interview, we also found that, even for people who were aware of the importance of stroke secondary prevention, they had little knowledge of how to perform physical activities or rehabilitation. For example, almost no patients knew the proper frequency and intensity of physical activities that they should achieve, nor the strategies to protect themselves from injury during exercise. In addition, walking was the most common way of exercise for most patients involved in the interview, and few patients knew how to train their upper limbs in their physical activities to promote their recovery of daily functions:

He [the physician] just told me to walk more.

Patient #2

I do not have other forms of exercise. I just walk for a while every morning in the village...I usually sit or sleep at home for most of the remaining day.

Patient #4

Therefore, we believe that providing more tips and guidance on how to undertake the guideline-recommended physical activity and rehabilitation will match the needs of stroke patients.

### Results From the Pilot Study

A total of 54 stroke patients from 4 villages in 2 townships in Nanhe County participated in the pilot study from February to May 2017. Among them, 51 participants (response rate: 94.4%) completed the feedback survey after the pilot study. Table 1 shows the characteristics of the participants. The average age of participants was 68.0 years (SD 9.2). Most of them were males (33/51, 65%), aged older than 65 years (33/51, 53%), married (40/51, 78%), with no education or having never graduated from primary school (27/51, 53%), had suffered ischemic stroke (45/51, 88%), and experienced more than 1 stroke event (31/51, 61%).

Among participants who responded to the feedback survey, 40 (40/51, 78%) and 43 (43/51, 84%) stated that they had successfully received text messages or voice messages during the pilot study, respectively. Table 2 shows the self-reported acceptance and understanding of messages for these participants. We observed a higher proportion of participants who were in favor of voice messages: 54% (23/43) of participants listened to the voice messages all the time, whereas only 35% (14/40) of participants self-reported their frequent text-message reading. Of 43 participants, 41 (95%) who listened to the voice messages self-reported that they could understand the content entirely or partially, whereas 30 of 41 (75%) participants reported that they were able to fully or partially understand the contents in the text messages. In addition, 39 of 43 (91%) participants who had ever received voice messages and 32 of 40 (80%) participants who had ever received text messages rated that the messages were helpful. In addition, 27 participants reported that their family caregivers had received text messages, and 11 of them reported that their family members had read the messages to them.

**Table 1:** Demographic characteristics and disease history of participants who responded to the feedback survey (N=51).

Characteristics				n (%)
**Demographic characteristics**				
		**Gender**		
			Male	33 (65)
			Female	18 (35)
		**Age group (years)**		
			≥65	33 (65)
			<65	18 (35)
		**Marital status**		
			Married	40 (78)
			Divorced, widowed, or unmarried	11 (22)
		**Education**		
			Never been to school	22 (43)
			Less than primary school	5 (10)
			Primary school	10 (20)
			Primary high school	10 (20)
			High school and above	4 (8)
		**Occupation**		
			Unemployed	7 (14)
			Self-employed including doing farm work	39 (77)
			Employed	4 (8)
			Others	1 (2)
		**Main caregiver**		
			Spouse	35 (69)
			Children	7 (14)
			Others	9 (18)
**Disease history**				
		**Stroke type**		
			Ischemic stroke	45 (88)
			Hemorrhage stroke	5 (10)
			Cannot remember	1 (2)
		**Suffered more than 1 stroke event**		
			Yes	31 (61)
			No	20 (39)

**Table 2:** Participants’ acceptance and perceptions of voice messages and text messages (of 54 participants, 51 completed the feedback survey, 40 responded that they had received text messages, and 43 responded that they had received voice messages).

Acceptance and perception		Voice messages (N=43), n (%)	Text messages (N=40), n (%)
**The frequency of reading/listening to the entire message**			
	All the time	23 (54)	14 (35)
	Sometime	20 (47)	14 (35)
	Never	0 (0)	12 (30)
**Level of understanding toward message contents**			
	Entire message	18 (42)	12 (30)
	A part of the message	23 (54)	18 (45)
	Cannot understand at all	2 (5)	10 (25)
**Whether think the messages were helpful**			
	Yes	39 (91)	32 (80)
	No	3 (7)	4 (10)
	Do not know	1 (2)	1 (3)
	Missing	—^a^	3 (8)

^a^3 participants who received text messages did not respond to this question. No missing values among participants who received voice messages on this question.

### Qualitative Results From the Pilot Study

After the pilot study, we interviewed 8 participants. Their feedback covered a variety of perspectives, including the content of the message, the form, and algorithms of message dispatching, and other barriers related to receiving and understanding the messages. This feedback guided our decision making in optimizing the message contents and sending the algorithm to fit the local context.

#### Message Contents

In line with the quantitative results, we also found that not all participants were able to understand the messages fully. Barriers to acceptance included the broad scope and complex content of the information covered in the messages, the relevance of messages toward their health conditions, the complexity of the terms and languages, and their own capacity to read and memorize information.

Many patients told us that they usually had poor memory and would easily forget things. They could usually understand the messages, but they would forget most of the contents after a short while if too much information was covered in a single message:

I could understand the message when I listened to it, but I would forget most of them quickly after I put down my phone.

Patient #6

In addition to the preference for simple information within a message, participants also reported that some terms in the messages were not in plain language, which made the messages more difficult to understand.

In terms of the impact of messages on helping them in managing the risk factors, some of the participants reported that not all the information was relevant to them. For example, messages related to smoking or drinking were only applicable to a small proportion of participants we interviewed:

He [the patient] drank every day before the stroke occurrence, but he quit after it. He doesn’t dare to drink anymore.

Spouse of Patient #2

#### Message Dispatch Form and Algorithms

Almost all patients who participated in the interview showed greater interest in and spoke highly of the voice messages, whereas very few participants reported that they had read text messages. This was mainly because of participants’ illiteracy in reading text or their technical capability in checking text messages on the phone:

My child bought a feature phone for me, and I know how to dial or pick up a call...I never read text messages. I am illiterate.

Patient #2

Participants also identified the potential factors that may facilitate their acceptance and understanding toward voice messages. Almost all participants involved in the interviews agreed that they preferred messages using the local dialect rather than standard Mandarin. Some of the participants also mentioned that a slower playback speed of the voice messages might improve their understanding of the message contents. In addition, participants recommended repeating the messages or extending the duration of messages so that they could better absorb the information when listening:

Sometimes I was doing my housework when I picked up the phone. She [the voice message] spoke too fast, and the message ended before I could concentrate my mind to listen to it.

Patient #4

In terms of the frequency and timing of message dispatch, most participants said that they were willing to receive the messages daily. In addition, participants suggested that receiving the message in the morning would be more helpful, especially for reminding them of taking medicines:

I often forget the medicines I should take in the morning because I usually have housework to do. But I usually can remember to take the medication at night before going to bed.

Patient #1

I would like to take the medicines, but I inevitably forget to do it sometimes... It would be, of course, good if someone can remind me every day.

Patient #2

#### Phone Scams

From our interview, we also found some other factors that may influence the implementation of the message-based intervention. Patients reported that they would reject phone calls from unknown numbers because they were afraid that it could be a phone scam. The numbers used to send the voice messages were different every day at the beginning of the pilot study, so some of the participants were cautious about the messages received and refused to listen to the voice messages. Participants mentioned that it would be easier for them to get the voice messages if the same number was used.

### Major Refinement of Messages

On the basis of the results from the pilot study, we made a series of decisions regarding the contents and dispatching algorithms of the messages to fit the local context and our target population better. Considering the prevalence of illiteracy and poor technical capacity in reading text messages, we decided to only use voice message delivery for the main study. Table 3 summarizes critical points where we optimized the message contents and algorithm.

To increase stroke patients’ understanding of the messages, we simplified the themes, structure, and language of each message. We highlighted the focus on medication adherence and physical activities that were appropriate for most stroke patients. Only a small proportion of the total messages were supplemented by general information related to stroke and management of other risk factors. In addition, we further refined each message by retaining only one key piece of information and used the same structure for each message (the program name, followed by a reminder sentence alternating between taking medicine and exercise regularly, and then specific health education information). The application of the health belief model [16] and the transtheoretical model on the stage of change [21] was carefully considered in refining each health education information and the order of messages. Furthermore, we invited physicians in the township hospitals to review all messages and amend any clinical or academic terms into plain language that would be commonly used and understood by the target population. Multimedia Appendix 2 shows some examples of the revised messages.

We also improved the message recording and dispatch algorithm to better suit the preferences of the target population. We continued to record voice messages using the local dialect and selected a slower playback speed, with the message being repeated twice per message delivery. In addition, we decided not to dispatch any text messages but rather send out voice messages daily. We changed the message dispatch time from 7 pm to 7 am so that the messages can better remind patients of medication taking. Furthermore, we optimized our message dispatch system so that a single consistent sender phone number would be displayed on participants’ phones.

**Table 3:** Comparison of key aspects of message optimization.

Aspects of message optimization		Pilot study	Main study
**Message content**			
	Themes	A broad scope of information, including management of metabolic risk factors, medication adherence, tobacco and alcohol control, dietary change, physical activity, and psychological recovery	Focused on medication adherence and physical activity and supplemented with other information on metabolic risk factor management and stroke in general
	Structure	Random structure for each message	Same structure for all messages: *program name + reminder sentence + health education information*
	Key information	Multiple key information within 1 message	Single key information for 1 message
	Languages	With some professional terms	Simple plain language
	Verification	By stroke specialists in first-tier hospitals	By village doctors and township physicians
	Health behavior change theory	Health belief model and the transtheoretical model (stage of change)	Health belief model and the transtheoretical model (stage of change)
**Message recording**			
	Speed	Normal speaking speed	Slower than the normal speaking speed
	Repeating	No repetition	Repeated once
	Dialect	Local dialect	Local dialect
**Message dispatch algorithms**			
	Text message	3 pm every 2 days	No text message
	Voice message	7 pm every 2 days	7 am every day
	Receiver	Patients and caregivers	Patients only
Senders		Random phone number	Single consistent phone number

### Messages Delivered Through System-Integrated Technology-Enabled Model of Care Trial

A message bank containing 92 voice messages was developed to support the SINEMA main trial. These messages were repeated for 4 times to support the year-long intervention. The voice messages had been delivered to more than 600 stroke patients who participated in the SINEMA trial in rural China since July 2017. During the year-long intervention, a total of more than 100,000 messages were sent to participants and some caregivers in the intervention arm if they had a phone and were willing to receive the messages.

## Discussion

### Principal Findings

In this study, we applied a multiple methodological study approach to develop mHealth messages to promote secondary prevention of stroke among community-dwelling stroke patients in rural China. We created the initial messages based on existing message banks from previous studies; involved patients in the initial study design in capturing their needs; evaluated patients’ acceptance through a 3-month pilot study, including quantitative surveys and in-depth interviews; and finally, refined and optimized the message contents and dispatching algorithm based on the feedback provided by participants.

This study is one of the few studies that have developed message-based intervention among people with cardiovascular diseases [25] and the first ever for stroke patients in rural China. Our study provides important information to researchers and policy makers about the feasibility of delivering voice message intervention for community-dwelling stroke patients in resource-limited settings. Our study further revealed that voice messages were favored over text messages among stroke patients in rural China, as voice messages do not require reading skills but only basic ability to receive phone calls. This finding is consistent with previous studies that demonstrated the preference for voice messages among the rural population in Bangladesh and Cambodia, who also had low rates of reading literacy [26,27]. Thus, we believe that voice messages have great potential to deliver interventions, especially among populations with low socioeconomic status.

Message-based interventions have been increasingly applied to promote chronic disease management. However, less attention had been paid to content design than its effectiveness. A number of studies have tested the effectiveness of phone-based interventions implemented among various populations worldwide [25,28,29], but only a few studies have described in detail the development process of message-based interventions and their lessons learned. Studies have found insufficient use of behavior change theories in mHealth messages and lack of involvement of the target population in the design process [30]. The modest effect and inconsistent results of message-based interventions suggest that we cannot overlook the design process. In our study, the health belief model [16] and the transtheoretical model (stage of change) [21] were embedded in the message contents by emphasizing the perceived threat of poor adherence to medication use, improving their perceived benefits in behavior change in line with the secondary prevention recommendation, providing them cues to action and encouraging their maintenance throughout different stages. In addition, considering the education level and health literacy of stroke patients in rural China, we did not involve our target population in the initial drafting of messages as some other studies did [31], but instead, we conducted interviews to understand their needs, tested their acceptance through a pilot study, and refined the messages based on their feedback.

Our study also revealed that the design of messages should adequately consider the local context and characteristics of the target audience. We found that message structure, language, complexity and relevance, and repetition are factors that influence patients’ acceptance and understanding. This finding is in line with some of the previous studies. For example, Muench and Baumel [32] analyzed the components of digital triggers in messages and differentiated triggers into 5 elements, including the sender, delivery approach, time of delivery, frequency, and trigger contents. Our study results also showed that a reliable sender, an accepted form of the message dispatch, optimal timing based on patients’ daily routine, and simple but relevant key messages were essential for the acceptance of message-based interventions. In addition, we also discovered certain factors that are specific to voice messages. For example, the speed of audio playback, the pattern of repetition, and the use of dialect may also improve audiences’ understanding of message contents [26].

### Limitations and Strengths

Our study has some limitations. First, in this pilot study, the acceptance of messages was measured using a self-designed short survey, rather than actual statistics from the message dispatch system in the number of messages received and reviewed. Thus, self-report bias could be introduced, as participants may have overrated their acceptance and understanding of messages when interviewed by village doctors because of recall or social desirability bias. In addition, although the questionnaire was designed based on previous studies in China, we did not conduct reliability and validity testing for the questionnaire; thus, we were not able to rule out all potential measurement errors from the study. Second, the participants involved in our contextual research and pilot study were conveniently sampled with a relatively small sample size based on set inclusion criteria. For example, more than 60% of our participants were male, and such proportion is higher than the proportion in general stroke patients, although the nationwide epidemiological study found a higher age-specific prevalence of stroke among men than that among women for the age groups of above 40 years [1]. Although participants may not be fully representative of all stroke patients in rural areas of China, we believe that the views they shared were common among stroke patients in rural China.

Our study also has several strengths. First, we have applied multiple methodologies, including literature review, expert consultation, qualitative in-depth interviews, and a pilot study, including a quantitative survey and personal interviews. By doing so, we were able to ensure a deeper involvement of the target population in the study design and gain a more comprehensive understanding of their needs, acceptance, and preferences. Second, the messages were developed by researchers and verified by health care professionals and village doctors. Such collaboration ensured that the messages were designed based on the clinical evidence and behavior change theories with conscientious consideration of local context. By applying these strategies, the contents of messages could be made reliable, feasible, and understandable. Third, the results of the initial design and pilot study were incorporated into the refinement of both the final content and delivery algorithm, potentially ensuring better acceptance and effectiveness of the message-based intervention.

### Conclusions

In summary, our study demonstrates the potential of using voice message interventions to improve chronic disease prevention and management among people with low education level in resource-limited settings. The development process indicates the importance of contextualization, which includes involving the target audience early and considering their preference and characteristics in the design process. The findings on the preference of participants for voice messages and the potential factors related to the acceptance add new evidence to the literature. These findings have general implications beyond stroke care in rural China and will be informative to other researchers who plan to develop message-based interventions for other disease conditions or in other research settings.

We recommend future studies to describe the message development procedures so that researchers could have better insight on the underlining mechanisms of the message design and the contextual environment that enabled the intervention implementation, which will facilitate the construction of the evidence based on the impact of the message-based interventions. In addition, more studies are needed to understand further the generalizability of the key aspects of message optimization that we found through our study among other populations. With such knowledge, we could better design the content and dispatching algorithms of the message-based interventions with higher adoption and maintenance.

## Appendix

Multimedia Appendix 1 Key searching terms, procedure, and findings from the literature review on the existing message banks.

Multimedia Appendix 2 Examples of messages.

## Abbreviations

mHealth: mobile health
SINEMA: System-Integrated Technology-Enabled Model of Care
