# Supplementary material for: Development and Local Contextualization of Mobile Health Messages for Enhancing Disease Management Among Community-Dwelling Stroke Patients in Rural China: Multimethod Study
Source: JMIR Mhealth Uhealth. 2019 Dec 17;7(12):e15758. doi: 10.2196/15758 (PMC6938591; doi:10.2196/15758)
Supplement: Multimedia Appendix 1 [file mhealth_v7i12e15758_app1.docx]

**Appendix 1. Key searching terms, procedure and findings from the literature review on the existing message banks**

### Key searching terms in PubMed

("Stroke"[Mesh] OR "Cerebrovascular Disorders"[Mesh] OR "Stroke"[Title/Abstract]) AND ("mhealth"[Title/Abstract] OR "mobile phone"[Title/Abstract] OR "smartphone"[Title/Abstract] OR "cell phone"[Title/Abstract] OR "SMS"[Title/Abstract] OR text messag*[Title/Abstract] OR "short message"[Title/Abstract])

### Inclusion and exclusion criteria:

- Inclusion criteria
- Population: Patients with stroke or cerebrovascular diseases or related risk factors
- Intervention: The message-based interventions (including voice messages, text messages) for patients to support their chronic condition management
- Message-bank: Mentioned about the design of the messages and had a message-bank available if required.
- Exclusion criteria:
- Studies not for patients with cardiometabolic diseases or studies designed for communication with healthcare providers only
- Interventions was built on mobile apps
- The study investigated on participants’ attitude or perceptions, rather than the real practice of the message development

### PRISMA diagram for the review of message banks


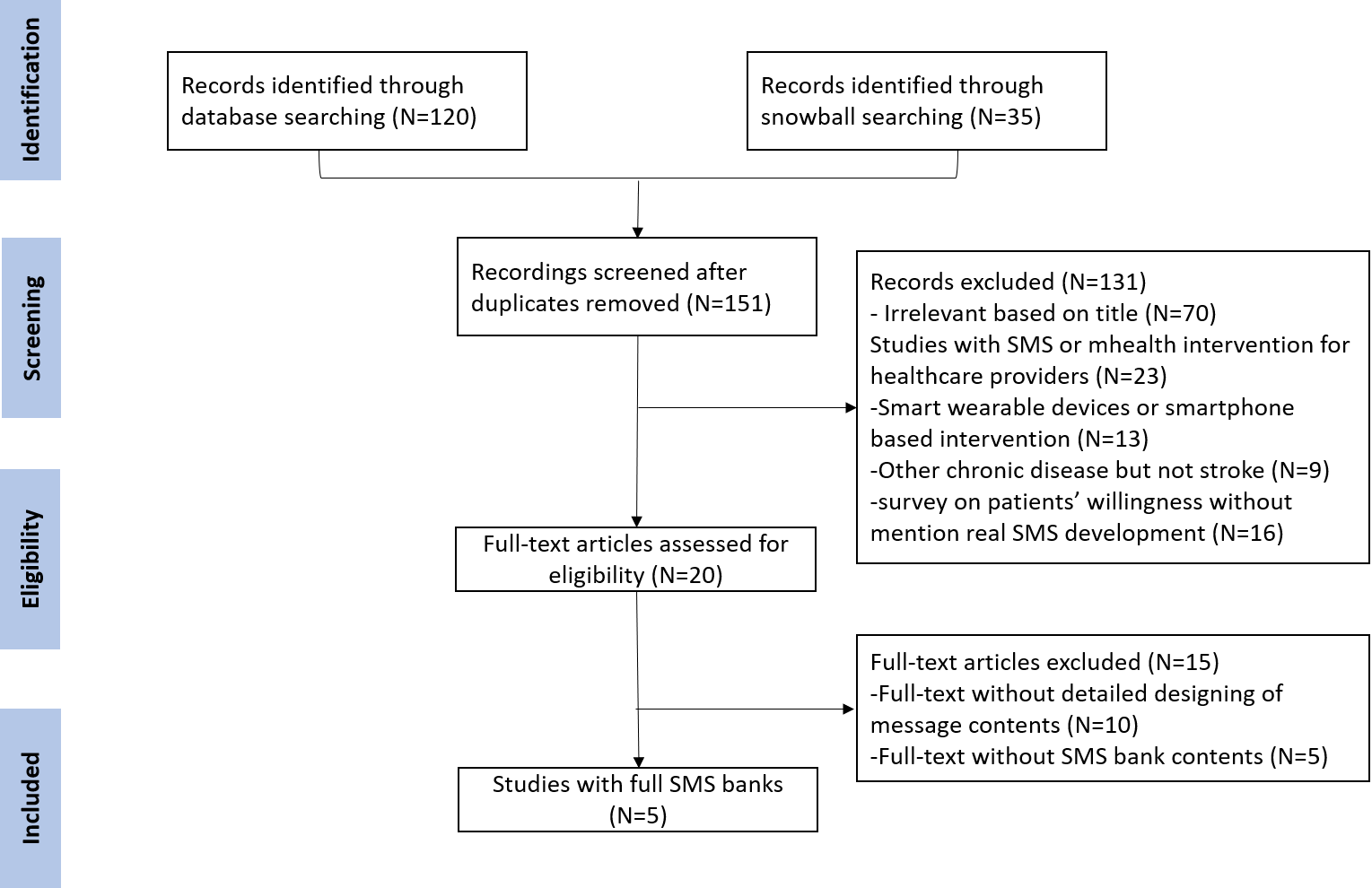


### Characteristics of existing message banks from the literature review

| **Study** | **Author (year)** | **Message**  **numbers** | **Objectives** | **Target population** | **Message contents** |
| --- | --- | --- | --- | --- | --- |
| 1 | Buis, L. R., et al. (2015) | 17 | To improve the quality of medication management through increasing medication adherence. | African Americans with uncontrolled hypertension | - Reminder for medication taking  - Knowledge about the DASH diet  - Satisfaction assessment  - Introduce of this study |
| 2 | Diez-Canseco, F., et al. (2015) | 64 | To conduct behavior change intervention for the primary prevention of hypertension in Latin American countries. | Low income, urban Spanish-speaking, aged 30-60 years old people living in or around the capital city of Argentina, Guatemala, and Peru | - salt and sodium intake  - fruit and vegetable intake  -consumption of high fat and sugar foods- Physical activity |
| 3 | Kamal, A. K., et al. (2015) | 51 | To increasing adherence to medications in patients with stroke. | Adult participants with a history of stroke longer than 1 month on multiple risk modifying medications | - Knowledge about stroke  - Importance of exercise and diet (modifiable risk factors)  - Effect and use of specific drugs of stroke  - Importance of optimal medication adherence |
| 4 | Bobrow, K., et al. (2016) | 16 | To motivate collecting and taking medicines and to provide education about hypertension and its treatment. | Adults who were diagnosed with hypertension and prescribed blood pressure–lowering medication | - Reminder for follow-up in hospital  - Reminder for medication taking  - Emotional supportive information on medication taking  - Knowledge about hypertension  - Tips for effectively taking medicine in time  - Information about healthy diet and exercise |
| 5 | Hacking, D., et al. (2016) | 90 | To improve health knowledge and self-reported health-related behaviors. | Hypertension clinic patients in a resource-poor setting in Cape Town, South Africa | -- Introduce of this study and greetings  - Knowledge of hypertension  -Information about healthy diet, mood, and exercise  - information about searching social support  - Reminder for follow-up in hospital |

### Characteristics of existing message banks from the literature review (Continued)

| **Study** | **Author (year)** | **Applied Behavior change theory** | **Message sending algorism** | **Language** |
| --- | --- | --- | --- | --- |
| 1 | Buis, L. R., et al. (2015) | Self-Regulation framework and the Health Belief Model | Participants customized number of text message reminders per day and the timing of dispatching | English |
| 2 | Diez-Canseco, F., et al. (2015) | The Transtheoretical Model (the Stages of Change Model) | sent weekly during a 12-month period | - Spanish (simple and direct language, understandable by laypeople from poor Latin American urban setting) |
| 3 | Kamal, A. K., et al. (2015) | Social Cognitive Theory, Health Belief Model and Michies Taxonomy of Behavioral Change Communication | sent twice per week at the time that patients were scheduled to take their medication as advised by their physician | English |
| 4 | Bobrow, K., et al. (2016) | A taxonomy model of behavior change techniques  (goals and planning, repetition and substitution, social support, and  natural consequence) | - Goal setting (outcome) were sent 48 hours prior  to scheduled appointment  - Review of behavior goals were sent 48 hours post scheduled Appointment  - The others were sent weekly | The SMS text messages used in the interventions were developed, translated, and tested in English, isiXhosa, and  Afrikaans, the 3 languages most commonly spoken by people living in Cape Town. |
| 5 | Hacking, D., et al. (2016) | The Health Belief Model | 5 SMS per week for a period of 17 weeks | The SMSs were translated from English into Xhosa (2 of the 3 official languages of the Western Cape) |

**Reference:**

Buis L R, Artinian N T, Schwiebert L, et al. Text Messaging to Improve Hypertension Medication Adherence in African Americans: BPMED Intervention Development and Study Protocol.[J]. Jmir Research Protocols, 2015, 4(1):e1-e1.

Diez-Canseco F, Zavala-Loayza J A, Beratarrechea A, et al. Design and Multi-Country Validation of Text Messages for an mHealth Intervention for Primary Prevention of Progression to Hypertension in Latin America[J]. Jmir Mhealth & Uhealth, 2015, 3(1).

Kamal A K, Shaikh Q N, Pasha O, et al. Improving medication adherence in stroke patients through Short Text Messages (SMS4Stroke)-study protocol for a randomized, controlled trial[J]. Bmc Neurology, 2014, 15(1):1-9.

Bobrow K, Farmer A J, Springer D, et al. Mobile Phone Text Messages to Support Treatment Adherence in Adults With High Blood Pressure (StAR): A Single-Blind, Randomized Trial.[J]. Circulation, 2016.

Hacking Damian, Haricharan Hanne J, Brittain Kirsty, et al. Hypertension Health Promotion via Text Messaging at a Community Health Center in South Africa: A Mixed Methods Study:[J]. JMIR mHealth and uHealth, 2016, 4(1).
