# Supplementary material for: Development and Local Contextualization of Mobile Health Messages for Enhancing Disease Management Among Community-Dwelling Stroke Patients in Rural China: Multimethod Study
Source: JMIR Mhealth Uhealth. 2019 Dec 17;7(12):e15758. doi: 10.2196/15758 (PMC6938591; doi:10.2196/15758)
Supplement: Multimedia Appendix 2 [file mhealth_v7i12e15758_app2.docx]

**Appendix 2. Examples of messages**

1. [“Care for stroke” project reminds you: have you taken your medicines today?] Do you know that the risk of stroke recurrence will increase if your blood pressure is above 140/90 mmHg? Therefore, please remember to take antihypertensive drugs to control your blood pressure. You should insist on taking antihypertensive drugs even if your blood pressure has been controlled under 140/90 mmHg. Long-term good medication adherence is important to the control of blood pressure.
2. [“Care for stroke” project reminds you: have you taken your medicines today?] Medicines do cost you a lot of money. However, the expenses of treatment will be much higher if you have a recurrent stroke. Only by taking medicines on time and living a healthy life can you prevent the stroke recurrence and save your money.
3. [“Care for stroke” project reminds you: have you exercised today?] Regular physical activities are beneficial for your rehabilitation. It is a good start to determine to exercise. Do not worry about that you do not have time to exercise. You can start from easy and simple physical activities. For example, you can start from a 15-minute walking every day and increase your walking time step by step based on your ability and physical conditions.
4. [“Care for stroke” project reminds you: have you exercised today?] After a few months of exercising, has your ability to walk increased? You can share your experiences of walking with your families and friends. Believe in yourself. If you can insist on exercising, your body will become healthier, which are good for your rehabilitation.
